# Lack of influence of GTP cyclohydrolase gene (*GCH1*) variations on pain sensitivity in humans

**DOI:** 10.1186/1744-8069-3-6

**Published:** 2007-03-07

**Authors:** Hyungsuk Kim, Raymond A Dionne

**Affiliations:** 1 National Institute of Nursing Research, National Institutes of Health, Bethesda, MD, USA

## Abstract

**Objectives:**

To assess the effect of variations in GTP cyclohydrolase gene (*GCH1*) on pain sensitivity in humans.

**Methods:**

Thermal and cold pain sensitivity were evaluated in a cohort of 735 healthy volunteers. Among this cohort, the clinical pain responses of 221 subjects after the surgical removal of impacted third molars were evaluated. Genotyping was done for 38 single nucleotide polymorphisms (SNPs) whose heterozygosity > 0.2 in *GCH1*. Influence of the genetic variations including SNPs and haplotypes on pain sensitivity were analyzed.

**Results:**

Minor allele frequencies and linkage disequilibrium show significant differences in European Americans, African Americans, Hispanic Americans and Asian Americans. Association analyses in European Americans do not replicate the previously reported important influence of *GCH1 *variations on pain sensitivity.

**Conclusion:**

Considering population stratification, previously reported associations between *GCH1 *genetic variations and pain sensitivity appear weak or negligible in this well characterized model of pain.

## Background

The role of 6(R)-t-erythro-5,6,7,8-tetrahydrobiopterin (BH4) in pain is suggested by the up-regulation of two of the three enzymes in the synthesis cascade of BH4 in the dorsal root ganglion following sciatic nerve injury [1]. GTP cyclohydrolase (GCH) catalyzes the rate limiting step, and sepiapterin reductase catalyzes the final conversion of 6-pyruvoyltetrahydropterin to the BH4. It was recently reported that single nucleotide polymorphisms (SNPs) in the gene encoding GCH (*GCH1*) alter both responses in healthy humans to noxious stimuli and the susceptibility of patients to development of neuropathic and inflammatory pain [2]. The authors also suggest a pain protective haplotype associated with the risk of developing persistent pain syndromes, which could be a useful tool to assess an individual's risk potential for chronic pain [2]. Based on this reported role of GCH in pain both in animals and humans, we investigated its contribution to genetic inter-individual variation in clinical pain sensitivity and analgesic responses. We have investigated the association between pain responses to experimental and clinical painful stimuli and genetic variations including SNPs and haplotypes of *GCH1*.

## Results

The minor allelic frequencies of each SNP in the *GCH1 *are shown in Table 1.

**Table 1:** SNPs genotyped in *GCH1*

Order	SNP ID	Location from transcription site	Nucleotide variation	Rarer allele frequency
1	rs8008858	-9,686	A/T	0.28
2	rs8007267	-9,462	G/A	0.28
3	rs2878172	-4,141	T/C	0.44
4	rs8017210	7,694	C/T	0.21
5	rs3783642	9,327	A/G	0.48
6	rs3783641	9,391	A/T	0.21
7	rs7147201	10,653	T/C	0.27
8	rs7147286	10,865	C/T	0.42
9	rs17128052	13,005	C/G	0.18
10	rs998259	14,499	G/A	0.14
11	rs10498471	15,813	C/T	0.22
12	rs8020798	16,162	G/A	0.21
13	rs8004018	18,834	T/C	0.25
14	rs8004445	18,864	C/A	0.25
15	rs3783639	20,864	A/G	0.20
16	rs3783638	21,157	C/T	0.26
17	rs3783637	21,412	G/A	0.18
18	rs10133650	23,257	G/C	0.47
19	rs12147422	25,515	A/G	0.25
20	rs17128050	25,651	A/G	0.17
21	rs7492600	32,655	C/A	0.26
22	rs2183081	32,779	T/C	0.47
23	rs8010282	39,275	T/C	0.21
24	rs9671455	40,450	G/C	0.26
25	rs9671371	40,895	G/A	0.33
26	rs2878169	43,537	C/A	0.06
27	rs12589758	43,668	T/A	0.22
28	rs12587434	43,947	A/C	0.23
29	rs7155309	46,679	C/T	0.27
30	rs2878168	48,745	A/G	0.22
31	rs4411417	48,967	A/G	0.22
32	rs10133662	53,260	T/C	0.39
33	rs10131232	53,622	C/T	0.40
34	rs752688	57,961	G/A	0.23
35	rs841	59,038	C/T	0.23
36	rs7142517	62,726	G/T	0.28
37	rs10483639	63,073	C/G	0.26
38	rs2057369	65,469	C/T	0.22

**Figure 1:**
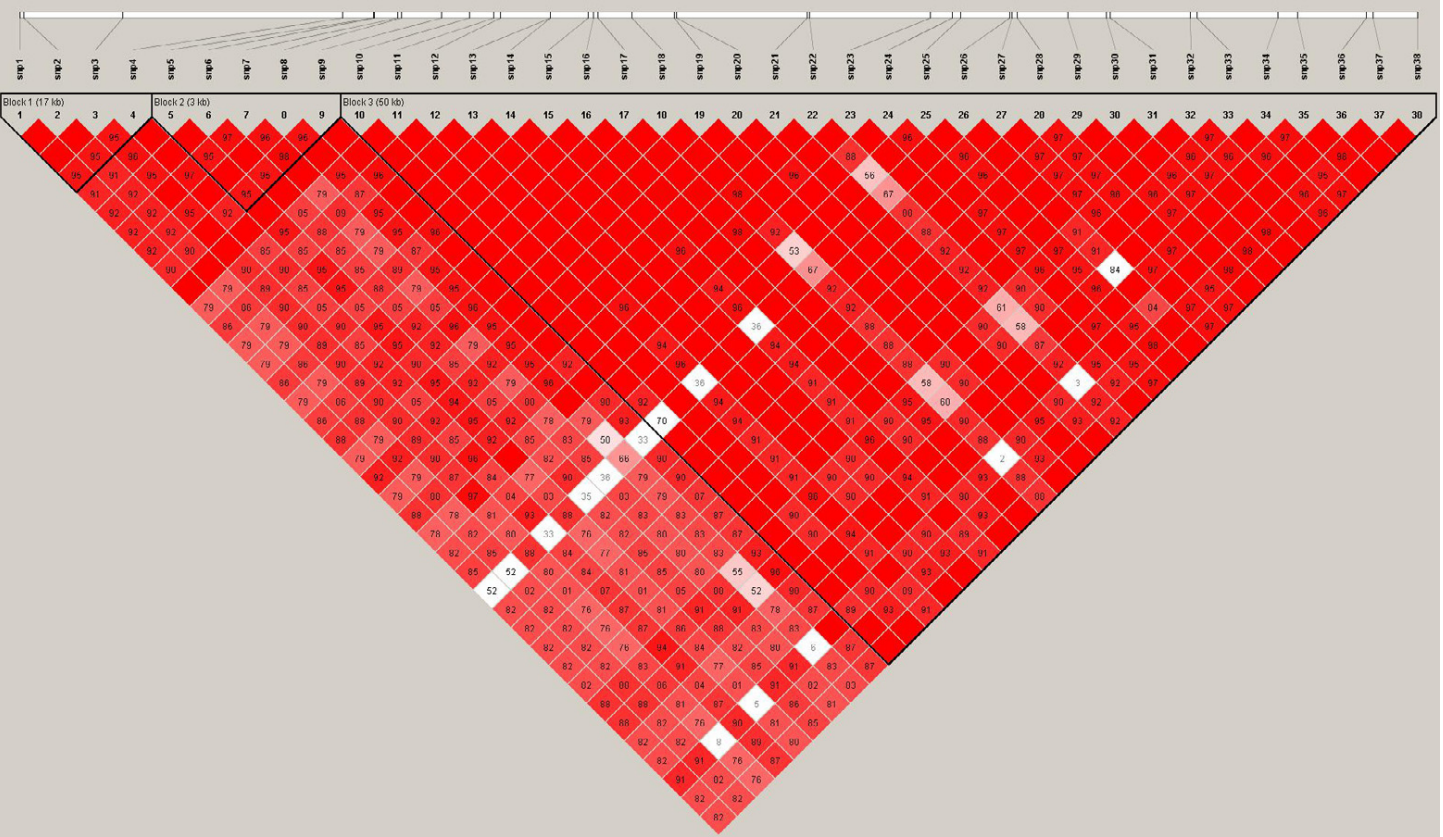
Linkage disequilibrium of *GCH1 *in European Americans (Haploview results with confidence interval method).

**Figure 2:**
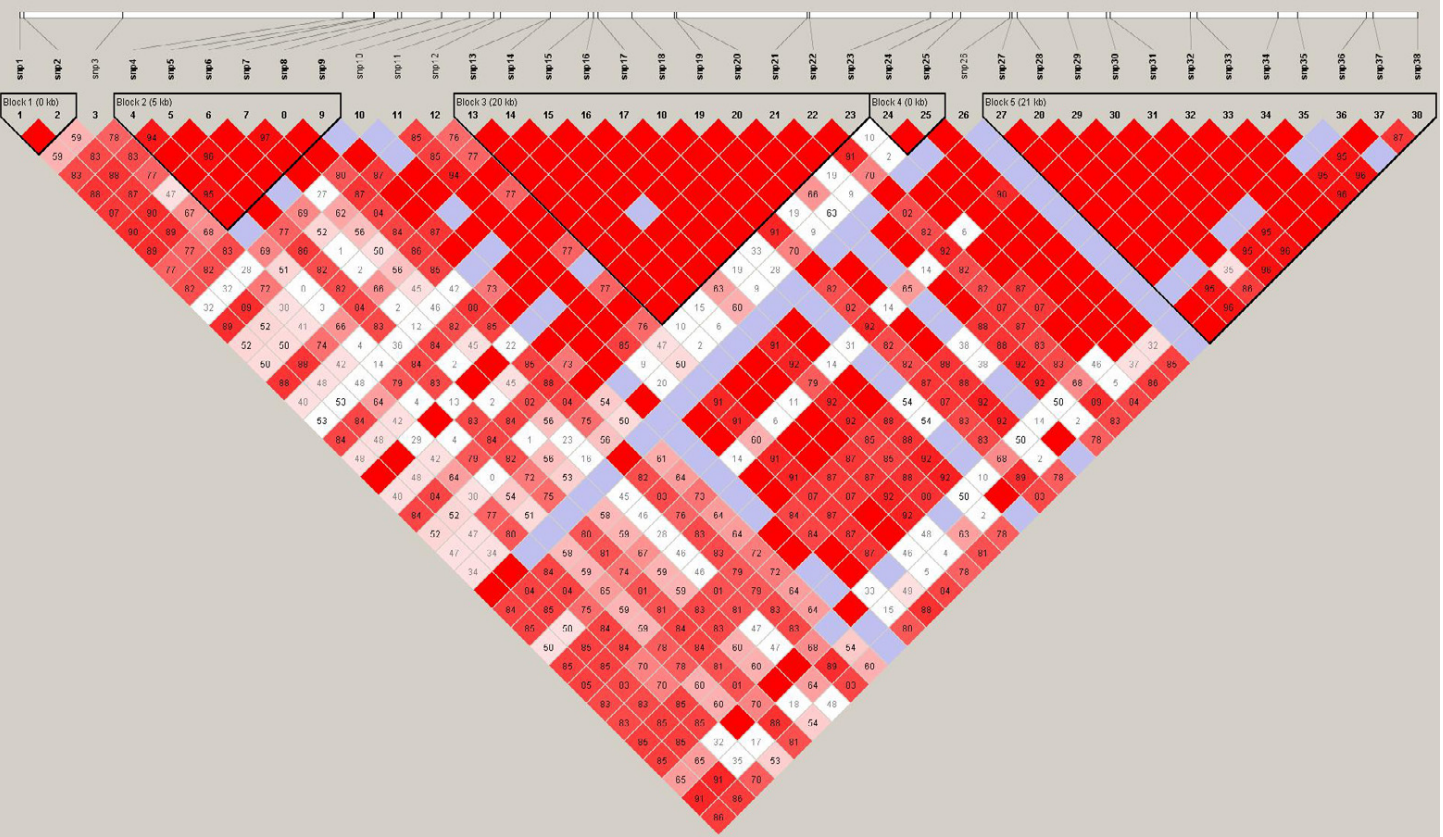
Linkage disequilibrium of *GCH1 *in African Americans (Haploview results with confidence interval method).

**Figure 3:**
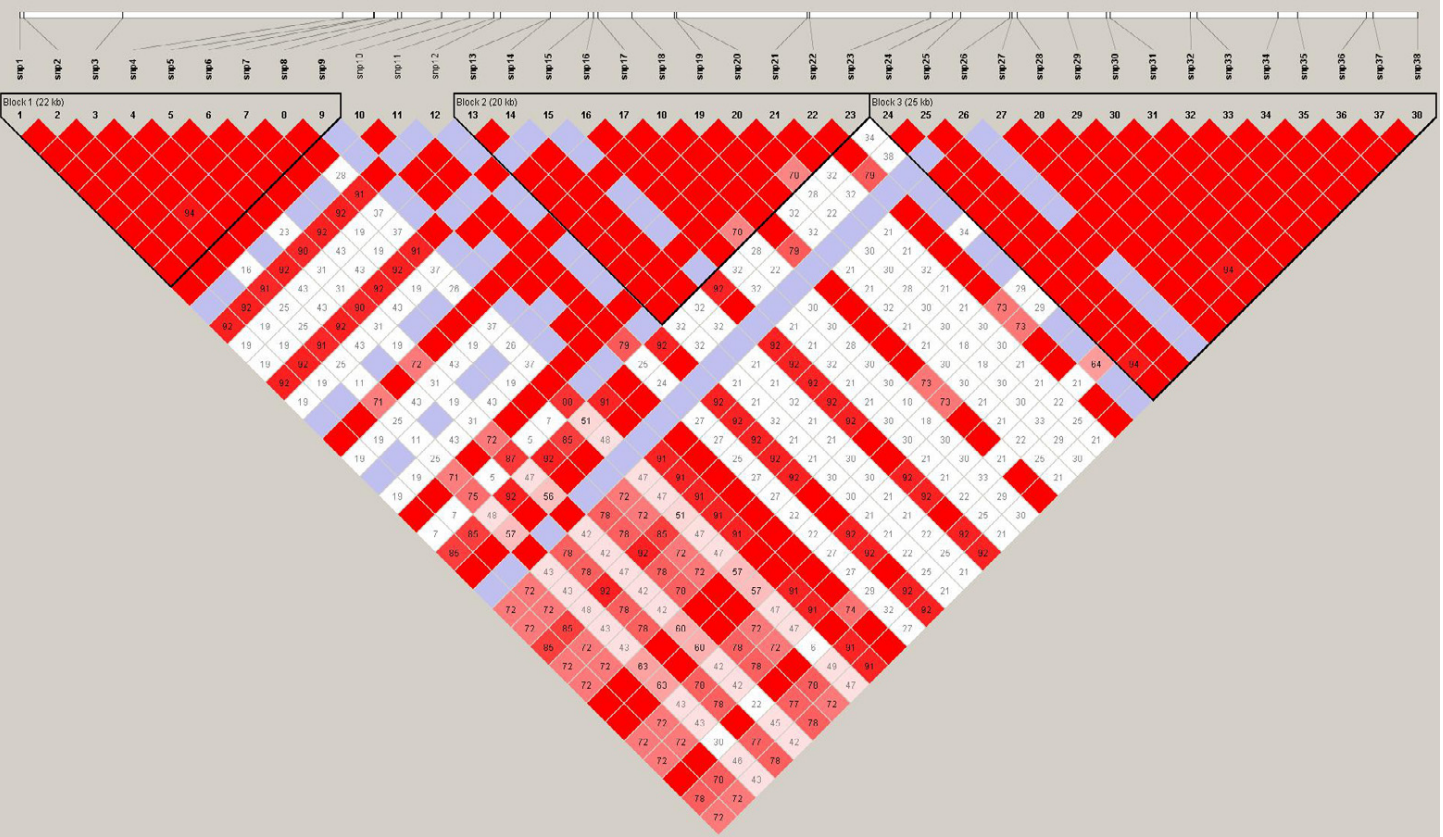
Linkage disequilibrium of *GCH1 *in Hispanic Americans (Haploview results with confidence interval method).

**Figure 4:**
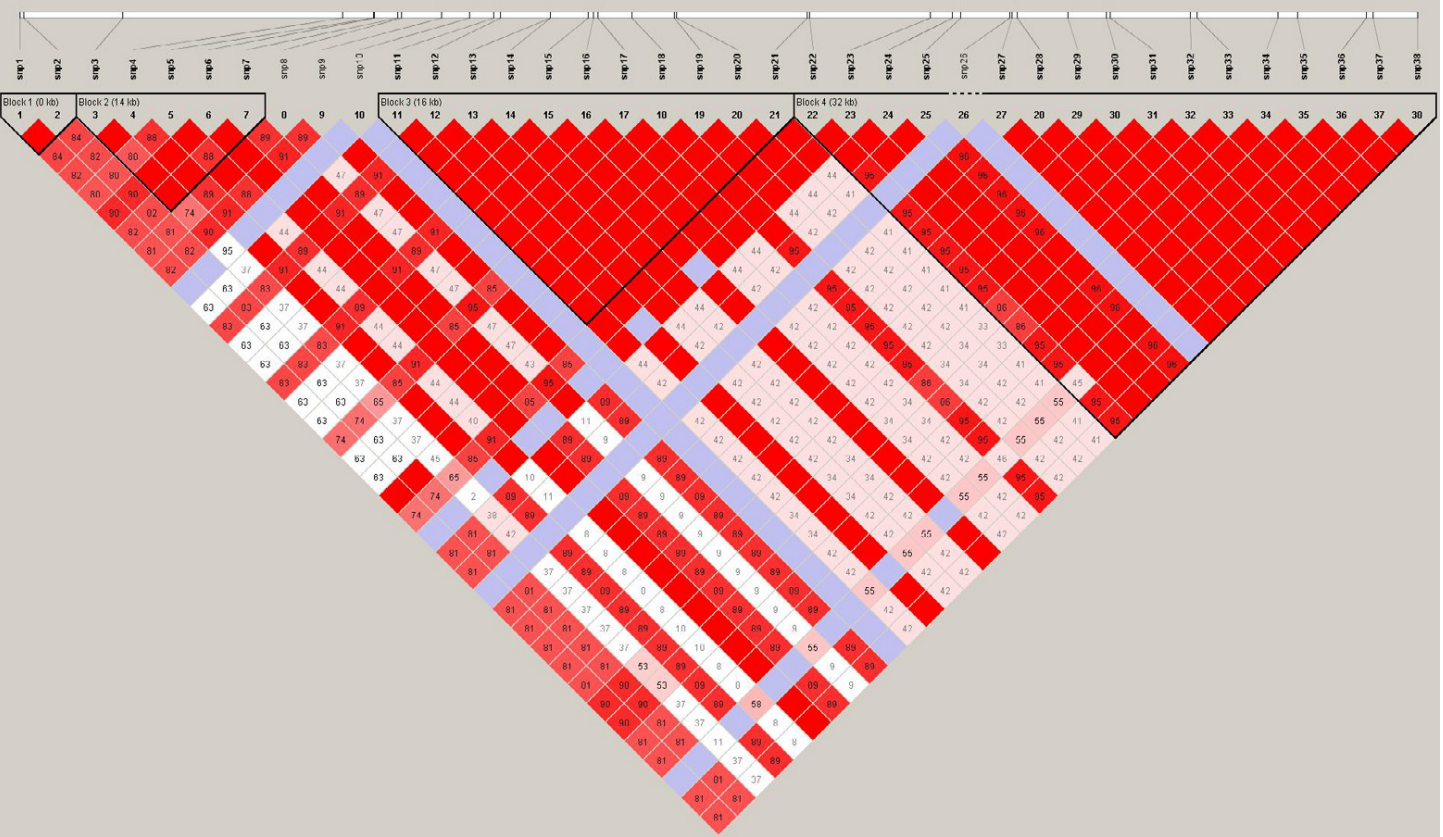
Linkage disequilibrium of *GCH1 *in Asian Americans (Haploview results with confidence interval method).

We next determined what haplotypes are formed by these SNPs. The total number of haploblocks found were 3 in European Americans (average minimum span = 24.0 kb) with 12 tag SNPs, 5 haploblocks with 21 tag SNPs in African Americans (average minimum span = 5.3 kb), 3 haploblocks with 14 tag SNPs in Hispanic Ameicans (average minimum span = 22.7 kb) and 4 haploblocks with 10 tag SNPs in Asian Americans (average minimum span = 16.1 kb). Position of those SNPs and haploblocks within the loci of *GCH1 *are shown in Figure 1, 2, 3 and 4. Their D' with confidence intervals and r^2 ^matrices in each ethnic population show large differences (see Additional file 1).

Further haploblock analysis was performed with European Americans only due to the small number of other ethnic populations. We found 3 haploblocks from *GCH1 *(4 SNPs spanning ~17.4 kb, 5 SNPs spanning ~3.7 kb and 29 SNPs spanning ~51.0 kb respectively) based on the confidence interval method.

In the *GCH1 *haploblock 1, a total of 5 haplotypes were detected for the 4 SNPs, with the most frequent haplotype (55.4%) composed of A_G_T_C. The 3 frequent haplotypes (> 5%) were 98% of total haplotypes. In the *GCH1 *haploblock 2, a total of 7 haplotypes were detected for the 5 chosen SNPs, with the most frequent haplotype (55.0%) composed of A_A_T_C_C. Four of the 7 haplotypes were frequent (> 5%), and these 4 haplotypes were 99% of the total haplotypes. In the *GCH1 *haploblock 3, a total of 17 haplotypes were detected for the 29 SNPs, with the most frequent haplotype (28.7%) and 5 frequent haplotypes were 88% of the total haplotypes. In the whole *GCH1*, 12 SNPs were tag SNPs European American population. Among 25 existing combinations of those 12 tag SNPs, the most frequent combination occupies 28.3% while 5 frequent (> 5%) combinations of tag SNPs are 87% of the total tag SNPs combinations (Table 2).

**Table 2:** Frequent haploblocks of *GCH1 *in European Americans

Haplotype		Frequency
Haploblock 1	A_G_T_C	0.55
	A_G_C_C	0.25
	T_A_C_T	0.17
	total	0.98
Haploblock 2	A_A_T_C_C	0.55
	G_T_C_T_G	0.18
	G_A_T_T_C	0.17
	G_A_T_C_C	0.09
	total	0.99
Haploblock 3	G_C_G_T_C_A_C_G_G_A_A_C_T_T_G_G_C_T_A_C_A_A_T_C_G_C_G_C_C	0.29
	G_C_A_T_C_G_C_G_C_A_A_C_C_T_C_A_C_A_C_T_G_G_C_T_A_T_G_G_T	0.19
	G_T_G_C_A_A_T_A_C_G_G_A_C_C_G_G_C_T_A_C_A_A_T_C_G_C_T_C_C	0.15
	G_C_G_T_C_A_C_G_G_A_A_C_T_T_G_A_A_T_A_C_A_A_C_T_G_C_G_C_C	0.06
	A_C_G_T_C_A_C_G_G_A_A_C_T_T_G_G_C_T_A_C_A_A_T_C_G_C_T_C_C	0.20
	total	0.88
Tag SNPs	A_T_C_A_A_C_G_C_G_G_G_G	0.28
	A_T_C_A_A_C_A_C_G_G_G_T	0.19
	T_C_T_G_T_T_G_C_A_C_A_G	0.16
	A_C_C_G_A_T_G_T_G_C_G_T	0.15
	A_C_C_G_A_C_G_C_G_G_A_G	0.08
	total	0.87

**Figure 5:**
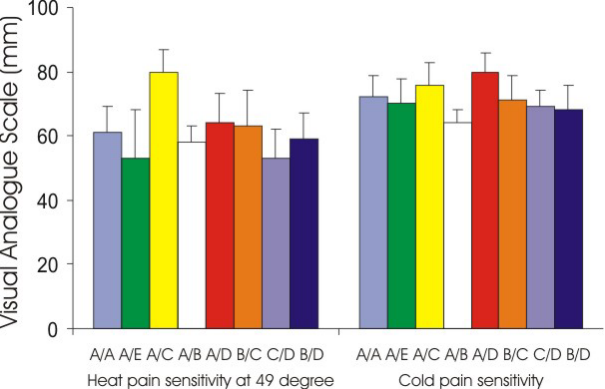
Non significant association between pain sensitivity and *GCH1 *tag SNP combinations in European American females. tag SNP combination A:A_T_C_A_A_C_G_C_G_G_G_G. B: A_T_C_A_A_C_A_C_G_G_G_T. C: T_C_T_G_T_T_G_C_A_C_A_G. D: A_C_C_G_A_T_G_T_G_C_G_T. E: A_C_C_G_A_C_G_C_G_G_A_G.

Considering the relatively dominating effect of gender on pain, male and female subjects were analyzed separately for the association between genetic variations (individual SNPs and combination of haplotypes) and responses to experimental and clinical painful stimuli. However, we could not find any significant genetic associations between variations of *GCH1 *including SNPs and haplotypes and pain sensitivity or analgesic responses in this cohort of European American females and males even without multiple testing corrections. Figure 5 shows an example of non-significant association between *GCH1 *genetic variations and pain sensitivity in European American females (*GCH1 *tag SNP combinations and heat and cold pain ratings).

## Discussion

We could not repeat any of the finding reported for humans by Tegeder et al. First, the linkage disequilibrium (LD) between SNPs and patterns of haplotype blocks in our sample are not consistent with their previous finding reporting one single block for *GCH1*. This inconsistency may be caused by the samples evaluated and the atypical interpretation of their data. The University of North Carolina and University of Florida cohorts of Tegeder et al. cite previous publications[3,4] with mixed population groups and leave open the question whether their present study is using mixed ethnicity groups as well. It has been shown that the ethnic demographics can have profound influence on LD between SNPs, haploblock structure, SNP frequency, and estimated effect size drawn from the data. It is for these very reasons that haplotype data are reported separately by ethnicity in the HapMap Project database.

When we analyzed haplotypes from our samples and the HapMap website within the same region using Haploview, we obtained different haplotype blocks between each ethnic groups, regardless of the algorithm used (implemented in Haploview, confidence interval, 4-gamete and solid spine method). Ethnic differences in the structure of haplotypes are consistent with other reports [5,6]. The single large 72 kb block identified by Tegeder et al for *GCH1 *with 168 Caucasian chronic lumbar root pain subjects is not consistent with the HapMap Caucasians (CEU), which may reflect a lack of generalizability for the Tegeder et al results. It is unclear how the data from the other cohorts is affected by one single large haploblock generated from the back pain study, especially when that one large block is not observed in the Phase II data released by the HapMap project. We found 3 haplotype blocks across *GCH1 *including its flanking region based on confidence interval method in European Americans and 5 blocks in African Americans with different size and different contributing SNPs. Hispanic Americans and Asian Americans also show unique haplotype blocks and LD values between each SNPs in *GCH1*. These results are similar to HapMap data and the general concept of smaller block size in samples of African ancestry.

Additionally, Tegeder et al proposed the low minor allele frequencies of a few markers as the cause of the disruption of the LD. However, almost 0 values of D' were obtained between 3 most frequent SNPs (10374 c>t as 29.69%, -4289 t>c as 37.42% and 3932 g>t as 35.76% in their minor allele frequencies) in supplementary figure 4 and supplementary table table 2 in Tegeder et al. The authors seem to interpret their data completely opposite direction. Unique haplotype patterns including block size, block numbers, contributing SNPs, tag SNPs along with LDs acquired in each ethnic population in our sample are consistent with HapMap data and clearly suggest that haplotype analysis with mixed population be avoided.

Using a mixed ethnic sample may also induce population stratification. This can cause false associations since the pattern of genetic variations as well as pain rating is not uniform between ethnic populations [7], requiring use of homogenous population in genetic association studies [8]. Considering significant differences of allele frequencies and pain sensitivities among ethnic populations, it would be more informative to analyze single ethnic origin population only compared to the total mixed sample to determine if the apparent association is due to population stratification.

Another possible reason of inconsistency is the pain phenotype. Because responses to different types of painful stimuli are genetically dissociable [9], *GCH1 *genetic variation may affect the responses to a specific type of painful stimulus, while not influencing other types of stimuli. However, Tegeder et al. used Z score for their analyses for a chimeric phenotype that combines pain threshold and pain tolerance, outcome measures at opposite ends of the sensory continuum. Use of an artificial outcome measure that has been transformed may not be an appropriate physiological representative of pain sensation and could potentially result in an artificial genetic association. Considering the non-significant association between *GCH1 *genetic variations and similar thermal pain sensitivity in our sample, the inconsistency cannot be easily explained by the differences in stimulus modalities. Additionally, the Z score is based on the assumption that the variables follow normal distribution, which pain as a phenotype does not [10]. Since the Z score is calculated by the mean and standard deviation, it would increase the risk of error when samples come from different means and standard deviations. Experimental heat pain threshold and tolerance have been reported that Caucasians have different means and standard deviations from African ancestry population.

It is also possible that our results may be false-negative. Many phenotypes with modest estimated genetic effects may result in false negative due to its underpowered studies and probably contribute to inconsistent replication. There are probably many common variants in the human genome with modest but real effects on common disease risk, requiring large samples to convincingly identify such variants [11]. Given these questions of study design related to population stratification, haploblock size, and disparate pain measures in Tegeder et al with similar statistical power of our samples, it is more probable that this association between *GCH1 *genetic variations and pain sensitivity are, if any, weak or negligible regardless of their strong in vitro and animal findings.

It is doubtful that a single gene accounting for large portion of the variability can explain the polygenic nature of pain. In many genetic association studies, true positive associations are rare, and most of the "significant" results rarely represent a true-positive association for which the genetic effect is accurately estimated [11]. Considering the overwhelming size of the human genome and relatively high risk of potential flaws in study design and analysis of complicated new methodology [12], replication of the association with independent population samples from studies strictly observing rules of genetic research is critical [13].

## Conclusion

Considering population stratification, previously reported associations between *GCH1 *genetic variations and pain sensitivity appear weak or negligible in this well characterized model of pain.

## Methods

### Subjects

Normal subjects (443 females and 292 males) were evaluated following informed consent under a human research protocol approved by the IRB. Demographics and characteristics of the cohort were previously described [14]. Briefly, subjects were not experiencing any clinical pain as symptomatic patients were referred elsewhere at the time of initial screening. Self reported ethnicity in the sample was 50.1% European American, 22.8% African American, 10.6% Hispanic, and 14.1% Asian American in composition. For the genotype linkage, we excluded individuals with mixed race parentage (2.3%).

Among 735 subjects, 221 patients underwent standardized surgery by the same oral surgeon removing third molar teeth that included at least one bony impacted mandibular third molar [15]. After receiving pre-medication with intravenous midazolam (4.9 ± 0.2 mg) and local anesthesia with 2% lidocaine (250.6 ± 43.0 mg) with epinephrine 1:100,000, a mucoperiosteal flap was raised and retracted, bone removed, and the teeth were sectioned as needed to facilitate extraction of the impacted lower third molars.

### SNP genotyping

For genotyping, 50 ml of venous blood from each subject was collected. DNA isolation was performed with the Puregene™ DNA isolation kit (Gentra Systems Inc., Minneapolis, Minnesota, USA) following manufacturer's instructions.

For SNP genotyping, Assays-on-Demand or Assays-by-Design SNP Genotyping Products (Applied Biosystems, Foster City, California, USA) were used. Each well contained 2.5 μl of Taqman universal master mix, 0.25 μl of genotyping assay mix and 2.25 μl of DNAse free water. Polymerase chain reaction (PCR) was performed under the following conditions: 95°C, 10 min followed by 40 cycles of 92°C, 15 seconds and 60°C, 1 minute in a Perkin-Elmer™ 9700 thermocycler (Perkin-Elmer Inc., Boston, Massachusetts, USA). Following PCR, fluorescence of each well was measured using the ABI Prism 7900 Sequence Detection System (Applied Biosystems, Foster City, California, USA). From the genomic sequences including their flanking regions, 38 SNPs (heterozygosity > 0.2, average distance = 2.0 kb) from *GCH1 *(genomic size = 75 kbs including flanking region) were screened. Detailed information of genotyped SNPs is in Table 1.

Genotype discrimination was performed using Taqman Sequence Detector version 2.1 software. Samples that failed to amplify were not included in the final analysis.

### Experimental pain sensitivity measurements

We measured pain sensitivity in response to experimental painful thermal stimuli and cold stimuli with separate visual analogue scale (VAS) ratings for pain intensity. Individuals were trained to use a sliding VAS by rating a visual gray scale. This procedure also provides an indication of each subject's comprehension of the rating process using a VAS [16]. For cold stimuli, we recorded cold pain intensity (CPI) VAS ratings every 30 seconds following submersion of the subject's hand up to the wrist into an insulated bucket filled with iced-water (2–4°C). We instructed subjects to keep their hand submerged while clenching and unclenching repeatedly to prevent local warming in the water until the pain reached an "unbearable level" or 180 seconds, whichever occurred soonest. The temperature of iced water was maintained by ice cubes separated from the subject's hand by a wire mesh. Subjects rated CPI at 30 seconds, if they withdrew their hand prior to 30 seconds, CPI was rated at their cold withdrawal time (CWT).

Individuals rated heat pain intensity (HPI) using a VAS following application of 35, 43, 44, 45, 46, 47, and 49°C thermal stimuli for 5 seconds. The thermode probe area was 1 cm in diameter and mounted in a housing to maintain constant pressure with the skin. The probe was self-applied at six different sites on the volar forearm within an area of ~40 × 100 mm. Four iterations were completed for each temperature by moving the probe from spot to spot to eliminate the possibility of sensitization or tissue damage. The order of each temperature was pre-determined for each trial, but the order was quasi-random to prevent subjects from anticipating each subsequent stimulus. Subjects were blinded with regard to the temperature of the stimulus.

### Clinical Pain measurement

Clinically induced pain was recorded with a paper and pencil form of a 100 mm VAS. After the extraction of the impacted third molars, pain was recorded every 20 minutes by VAS until subjects requested analgesic medication as the local anesthesia was eliminated and post-operative pain onset occurred. Ketorolac tromethamine (Toradol) was administered intravenously at the recommended dose (30 mg) and pain was recorded by VAS again at 15 minutes interval for 180 minutes. The maximum post-operative pain rating, onset time of post-operative pain, the analgesic onset time after medication and pain relief at 180 minutes after medication were used as measures of clinical pain.

### Data analysis

To resolve phase unknown genotypes and estimate population frequencies in unrelated individuals we employed PHASE method, a probability based Bayesian algorithm. Haploblocks based on confidence interval rule [5] were generated by Haploview version 3.11. To quantify linkage disequilibrium (LD), widely used D' and r^2 ^were calculated. Both measures are built on the basic pairwise-disequilibrium coefficient, D, which is the difference between the probability of observing two marker alleles on the same haplotype and observing them independently in the population. A value of 0 implies independence, whereas 1.0 means complete co-transfer.

Due to the smaller sample size when subdivided into ethnic groups, second association analyses with experimental and clinical pain was done only in European Americans. For the associations between haplotypes, individual SNPs and cold/heat pain sensitivity and/or clinical pain responses, statistical evaluation was performed with analysis of variance (ANOVA) models. Groups of frequency less than 5% were excluded for the association analyses.

## Competing interests

The author(s) declare that they have no competing interests.

## Authors' contributions

HK carried out the molecular genetic studies, participated in the DNA extraction, genotyping, statistical analysis and drafted the manuscript. RD conceived of the study and participated in its design and coordination and helped to draft the manuscript. All authors read and approved the final manuscript.

## Supplementary Material

Additional File 1 D' with confidence intervals and r^2 ^matrices. D' with confidence intervals and r^2 ^matrices between SNPs in European Americans, African Americans, Hispanic Americans and Asian Americans. Click here for file
